# An Assay to Study Intra-Chromosomal Deletions in Yeast

**DOI:** 10.3390/mps2030074

**Published:** 2019-08-26

**Authors:** Bailey E. Lucas, Matthew T. McPherson, Tila M. Hawk, Lexia N. Wilson, Jacob M. Kroh, Kyle G. Hickman, Sean R. Fitzgerald, W. Miguel Disbennett, P. Daniel Rollins, Hannah M. Hylton, Mohammed A. Baseer, Paige N. Montgomery, Jian-Qiu Wu, Ruben C. Petreaca

**Affiliations:** 1 Department of Molecular Genetics, The Ohio State University, Marion, OH 43302, USA; 2 Microbiology Program, The Ohio State University, Columbus, OH 43210, USA; 3 Molecular Genetics Program, The Ohio State University, Columbus, OH 43210, USA; 4 Department of Molecular Genetics, The Ohio State University, Columbus, OH 43210, USA

**Keywords:** DNA double-strand breaks, Genetic Recombination, Yeast

## Abstract

An accurate DNA damage response pathway is critical for the repair of DNA double-strand breaks. Repair may occur by homologous recombination, of which many different sub-pathways have been identified. Some recombination pathways are conservative, meaning that the chromosome sequences are preserved, and others are non-conservative, leading to some alteration of the DNA sequence. We describe an in vivo genetic assay to study non-conservative intra-chromosomal deletions at regions of non-tandem direct repeats in *Schizosaccharomyces pombe*. This assay can be used to study both spontaneous breaks arising during DNA replication and induced double-strand breaks created with the *S. cerevisiae* homothallic endonuclease (*HO*). The preliminary genetic validation of this assay shows that spontaneous breaks require *rad52^+^* but not *rad51^+^*, while induced breaks require both genes, in agreement with previous studies. This assay will be useful in the field of DNA damage repair for studying mechanisms of intra-chromosomal deletions.

## 1. Introduction

The inappropriate repair of DNA double-strand breaks (DSBs) can cause different forms of structural chromosomal instability such as translocations, deletions, duplications and inversions [1,2]. These aberrations are the types of genomic instability seen in cancer cell karyotypes [3].

There are two main sources of DNA double-strand breaks—endogenous and exogenous. Both sources can produce similar types of lesions and are repaired by the same mechanisms [4]. Replication stress is the greatest producer of endogenous breaks. Replication forks may stall as they run into hard-to-replicate heterochromatin structures [5,6] or during collisions with RNA polymerases [7]. Such replication stalls are rescued by the recombination machinery which has evolved precisely to deal with stress arising from the replication of long genomes [8]. Exogenous breaks are caused by environmental chemicals or radiation.

DNA DSB repair occurs by two distinct genetic pathways: homologous recombination (HR) or non-homologous end joining (NHEJ) [4]. HR uses an intact template sequence to copy the missing/broken region and requires the RAD52 epistatic group (RAD52, RAD51, RAD54, RAD55/57 as well as several accessory nucleases and helicases) [9,10]. NHEJ requires Ku70/80 and Lig4, and involves the localized repair of breaks with no major sequence rearrangements [11]. In yeast, as in humans, the two repair pathways are cell-cycle regulated under the control of the cyclin-dependent kinase with NHEJ, acting primarily in G1 and HR in S-phase and mitosis [12,13,14,15,16,17]. Furthermore, NHEJ antagonizes HR as Ku70/80 inhibits the HR pathway in G1 [15].

Several in vivo assays have been designed in various model systems, as well as in human cells, to study the genetic requirements for DSB repair (we only reference a few) [18,19,20,21,22,23,24,25,26]. Such studies have contributed vastly to our understanding of how chromosomal instability arises. We also previously briefly described an assay to study chromosomal instability at non-tandem direct repeats arising from spontaneous damage [27]. However, this assay was only briefly characterized and not sufficiently validated. Here, we present an improved assay that can be used to study both random and induced DSBs and describe the protocol for this method. We also provide a preliminary characterization of the genetic requirements for repair of these different types of breaks. Our results show that induced breaks are likely to be repaired by different mechanisms than spontaneous breaks, in agreement with what has been previously shown. This assay should become a valuable tool in the field of yeast genetics to study intra-chromosomal deletions.

## 2. Experimental Design, Methods and Materials

**Figure 1:**
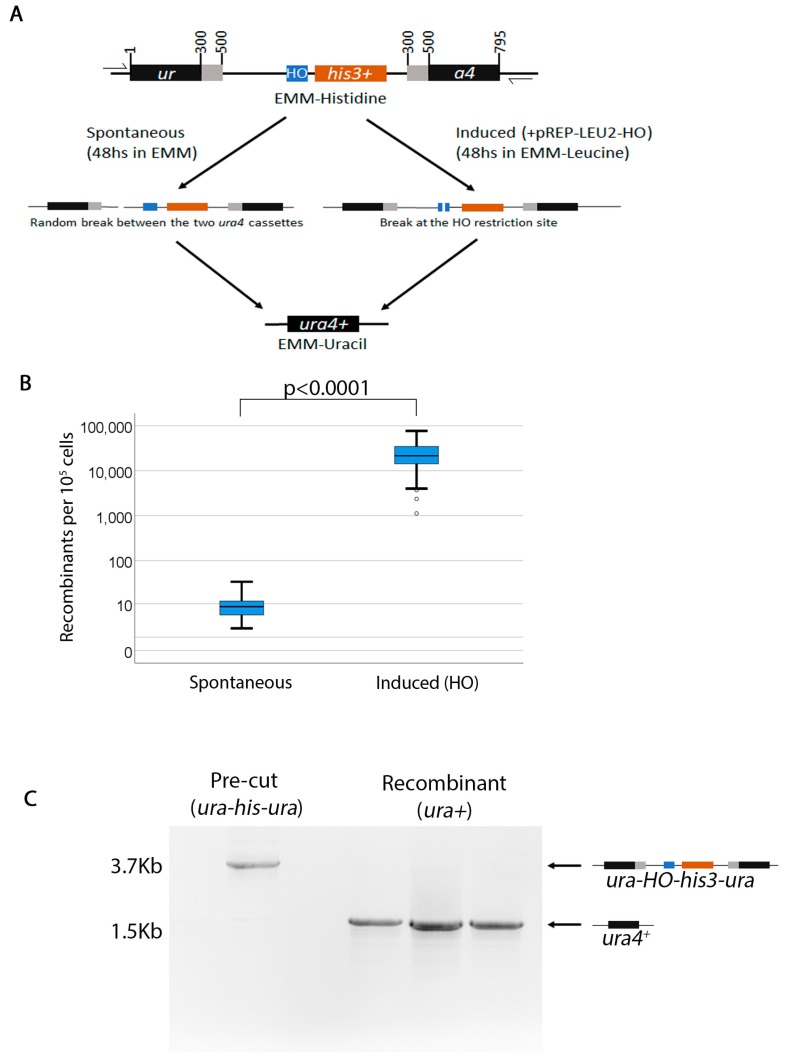
An assay to study spontaneous and induced double-strand breaks at regions of non-tandem repeats. (**A**). The *ura-his-ura* assay. In this assay, two non-functional *ura4* alleles flank a functional *his3^+^* allele. The *ura4* alleles have 200 bps of identical overlapping sequences, creating two non-tandem repeats (gray areas). The *S. cerevisiae* homothallic endonuclease (*HO*) is cloned just upstream of the *his3^+^* gene. The *HO* enzyme is on a *LEU2* plasmid under the control of the *nmt1* promoter which can be repressed with thiamine. Spontaneous *ura4*^+^*his3*^−^ recombinants are assayed by growing cells in EMM+UraHisAdeLeu media for 48 h then plating on selective EMM-Uracil. Induced break recombinants are assayed by growing cells for 48 h in media without thiamine to de-repress the *HO* endonuclease, while maintaining selection for the plasmid (EMM-Leucine). Cells are then plated on EMM-Uracil. All experiments were performed at 32 °C. (**B)**. Box plot showing the frequency of recombinants for both induced and spontaneous breaks. (**C)**. PCR across the *ura-his-ura* cassette in both pre- and post-recombination strains. Half arrowheads in (**A**) show approximate positions of primers.

We previously reported an assay that monitors chromosomal instability [27]. In this assay, two non-functional *ura4* fragments were placed on either side of a functional *his3^+^* gene (Figure 1A). The *ura4^+^* fragments contain 200 bp of overlapping identical sequences (gray region), creating two non-tandem repeats (referred to from here on as the *ura-his-ura* cassette). Here, we improved on this assay by introducing the *S. cerevisiae* homothallic endonuclease (*HO*) restriction site next to the *his3^+^* gene. The endonuclease restriction site is identical to that described in [28]. Primers 5′-ggaattcggccaggtacctttcagctttccgcaacagtataaagtactctgca-3′ and 5′ gagtactttatactgttgcggaaagctgaaaggtacctggccgaatcctgca-3 with *Pst*I restriction site overhangs were used to amplify the HO endonuclease restriction site. The PCR was cloned into the *Pst*I site of plasmid pRCP16 described previously [27] to generate pRCP20. The *ura-his-ura* cassette was released with *Sac*I and *Kpn*I and transformed into FY1828 to create RCP24. Unlike in our previous report where we studied recombination in the centromere heterochromatin, here we introduced the *ura-his-ura* cassette at the endogenous *ura4^+^* locus (Chr.III 116575-115781). The *ura4^+^* was replaced by the cassette. Thus, this strain is *ura* auxotrophic and *his* prototrophic. This assay can monitor both random breaks that may arise during DNA replication and *HO*-induced breaks. The *HO* endonuclease is expressed from the p*REP81X-HO* plasmid [29]. p*REP81X* was used as vector control.

### 2.1. Spontaneous Break Recombination Protocol

1. Streak cells onto EMM-Histidine plates from the −70 °C freezer. Grow at 32 °C for 3–4 days until colonies appear. Although the cassette is quite stable, to ensure that starting cells are *ura*^−^*his*^+^, it may be necessary to replica plate the EMM-His onto 5-FOA and choose only those colonies that grow on both plates.
2. Resuspend 10 colonies each in 100 μL water in microtubes, count cells and release in 4 mL liquid EMM+UraHisLeuAde at 100 cells/microliter. Incubate tubes at 32 °C in the rotator for approximately 48 h.
3. Determine the concentration of the cells in the tubes by counting cells using a hemocytometer and plate onto EMM-Uracil+Phloxin B at 10^5^–10^6^ cells per plate. Because *ura^−^* cells tend to cannibalize themselves, sometimes false positives appear. The addition of Phloxin B makes it easier to identify false positive because it stains *ura^−^* cells bright red. Phloxin B does not have an effect on recombination rate (Supplementary Tables S2 and S3). Furthermore, we recommend using large 150 mm × 15 mm plates particularly when plating at higher density. Plate a YES control as well for each colony plated on EMM-Uracil at 1000 cells per plate. This control is important to check for cell viability and accuracy in counting. Although we used YES for this control, a better control may be EMM+Uracil+Histidine. This maintains consistency with the experimental plate which is EMM not YES.
4. Incubate all plates at 32 °C until colonies appear—usually 3%#x2013;5 days for WT and longer for mutants.
5. Count colonies on both the YES control and EMM-Uracil plates and record the numbers. Although Phloxin B allows for easier differentiation of *ura4+* prototrophic colonies, to ensure that all colonies on the EMM-Uracil plates are in fact Ura^+^, this plate can be replica plated onto 5-FOA. All *ura4+* colonies that grow on EMM-Uracil should die on 5-FOA.

### 2.2. Induced Break Recombination Protocol

1. Streak cells onto EMM-Leucine+Thiamine plates from the −70°C freezer. Incubate at 32 °C for 3–4 days.
2. Resuspend 10 colonies in water, count cells and release in 4 mL liquid EMM-Leucine at 100 cells/microliter. Incubate tubes at 32 °C with rotation for approximately 48 h.
3. Determine the concentration of the cells in the tubes and plate onto EMM-Uracil (100 mm × 15 mm plates) at 10^4^ cells per plate. Plate on YES as well at 10^3^ cells per plate.
4. Incubate all plates at 32 °C until colonies appear.
5. Count colonies on both YES and EMM-Uracil plates and record the numbers.

### 2.3. Characterization of the Assay

Spontaneous *ura4^+^ his3^−^* recombinants arise at an average frequency of approximately 1 in 10^4^ cells (Figure 1B). As expected, when the break is made by the endonuclease, the frequency is much higher (2.5 in 10 colonies) (Figure 1B). This assay does not appear to report conversion (e.g., *ura4^+^ his3^+^* colonies). PCR analysis of several recombinants with primers flanking the *ura4^+^* ORF showed that the *ura-his-ura* cassette has been converted to *ura4^+^* (Figure 1C). Sporadically, we did find some colonies that were *ura4^+^ his3^+^*, which appeared at a much lower frequency and only when we induced the break (Supplemental Figure S1A). To understand what these *ura4^+^ his3^+^* colonies were, we used PCR to check the size of the locus in the *HO*-induced recombinant colonies (Supplementary Figure S1B). When primers flanking the *ura4^+^* ORF are used, we found that both the *ura4^+^* and the *ura4^+^ his3^+^* are the same size—indicating that both are deletion outcomes. Next, we checked whether the *ura4^+^ his3^+^* colonies arose as a result of gene conversion between the *his3^+^* in our assay and the *his3-D1* locus [30]. PCR across the *his3^+^* locus showed that the *his3-D1* deletion is present in both the *ura-his-ura* (pre) and the recombinant *ura4^+^ his3^+^* colonies, suggesting that the *his3-D1* allele has not been converted to *his3^+^* (WT) (Supplementary Figure S1C). However, PCR with primers within the ORF *his3^+^* detected the presence of the *his3^+^* ORF in the *ura4^+^ his3^+^* strains. We concluded from this PCR analysis and the very low frequency of the *ura4^+^ his3^+^* recombinants that the *his3^+^* must arise due to some spurious integration of the ORF elsewhere in the genome. Thus, this assay can be primarily used to test deletions.

### 2.4. Strains

The strains used in this study are listed in Supplementary Table S1. The construction of the *ura-his-ura* recombination cassette has been described previously [27]. This cassette has been slightly modified by cloning the *S. cerevisiae HO* endonuclease restriction site immediately upstream of the *his3^+^* gene, as described above. The *HO* restriction site sequence is identical to that described in [28]. Standard yeast genetics have been used to cross the recombination mutants with the *ura-his-ura* cassette.

### 2.5. PCR Analysis

The primers for the PCR in Figure 1C were, 5′-agctacaaatcccactggct-3′ and 5′-tgatattgacgaaacttttt-3′. PCR was performed using Phusion^®^ High-Fidelity DNA polymerase (NEB) and GC buffer at 55 °C annealing temperature (34 cycles).

### 2.6. Data Analysis

For all assays, the data were adjusted for viability and error in plating using the numbers on the YES plates (# colonies EMM-Uracil/(# colonies on YES/1000). The number “1000” represents the colonies intended to be plated on the YES plate. For example, if only 900 colonies appeared on the YES plate, then 900/1000 = 0.9 efficiency of plating. This means that a 10% error was made and the division of EMM-Uracil colonies by 0.9 normalizes the numbers to 100% plating efficiency. Thus, the YES plate serves as a control for both viability and plating errors and normalizes all experiments. This normalization was also important in order to control for systematic errors that might have been introduced as different people did the experiments, and is not unlike normalizations used previously [27,31]. The resulting value was then multiplied by the dilution factor so that the results were normalized to a recombination frequency of 10^5^ for spontaneous breaks and 10^4^ for induced breaks. Descriptive statistics and graphs were generated using SPSS.

## 3. Genetic Validation of the Assay and Discussion

### 3.1. Analysis of Spontaneous Breaks

**Figure 2:**
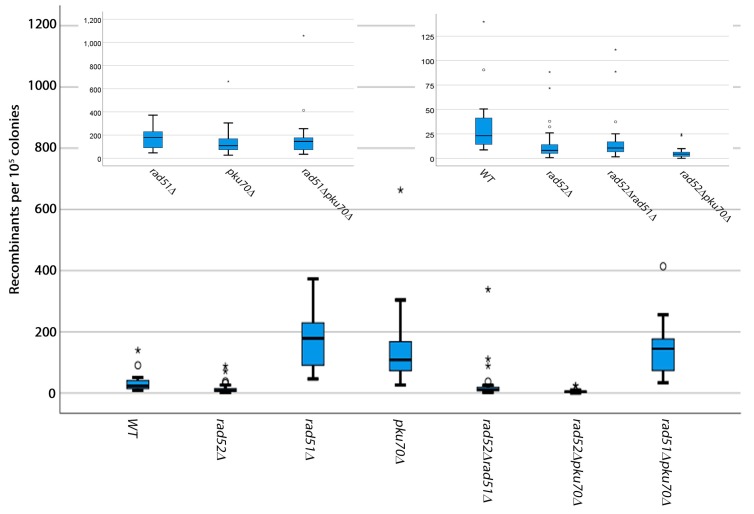
Genetic requirements for spontaneous breaks. Box plots showing the spontaneous recombination frequency per 10^5^ colonies. Cells were grown on EMM-Uracil plates for 3–5 days at 32 °C. For clarity, insets are shown for strains with similar recombination frequencies.

We next carried out some preliminary characterization of the genetic requirements for these deletions (Figure 2). We found that *rad52^+^* is required for spontaneous breaks but *rad51^+^* and *pku70^+^* are not (Figure 2, Supplementary Tables S2 and S3). In fact, both *rad51^+^* and *pku70^+^* appeared to inhibit deletion outcomes. These results suggest that spontaneous break repair relies on a pathway that requires both *rad51^+^* and *pku70^+^*. *rad51^+^* has been previously shown to suppress chromosomal rearrangements in *S. pombe* arising from improperly repaired spontaneous breaks [25,32]. *rad51^+^* is also not required for deletion outcomes, but is essential for gene conversion [33]. This indicates that spontaneous break repair is initially channeled through a homologous recombination pathway that requires *rad51^+^*, presumably by attempting to initiate a crossover. However, this pathway is not very efficient, most likely because the break occurs between direct repeats—a process which favors repair by single-strand annealing. Mechanisms of repair of spontaneous breaks by single-strand annealing that do not rely on *rad51^+^* have been proposed in *S. pombe* [34].

The loss of *pku70^+^* also increases recombination outcomes arising from spontaneous breaks, indicating that *pku70^+^* suppresses these deletions as well. Recent evidence in *S. pombe* shows that *pku70^+^* controls resection at stalled replication forks [35]. The loss of *pku70^+^* leads to an increase in the resection tract but a decrease in Rad51 binding. These two events combined suggest that *pku70^+^* and *rad51^+^* work in the same pathway and in the repair of direct repeats in our assay. This repair pathway most likely occurs through single-strand annealing when either *pku70^+^* or *rad51^+^* is lost.

In higher eukaryotes and fission yeast, *rad52^+^* is not essential for all forms of homologous recombination repair [36,37]. The fact that some repair still occurs in the absence of *rad52^+^* indicates that, at a low percentage, *ura4^+^* may be reconstituted by some other form of repair that does not rely on *rad52^+^.* These genetic results validate that this assay reports deletions only.

Sometimes the deletion of *rad52^+^* in *S. pombe* acquires a suppressor that attenuates the sensitivity of the strains to DNA-damaging drugs. However, these results are not due to the effect of such a suppressor, because all strains used here are still sensitive to methyl–methanosulfonate (MMS) (Supplementary Figure S2).

### 3.2. Analysis of Induced Breaks

**Figure 3:**
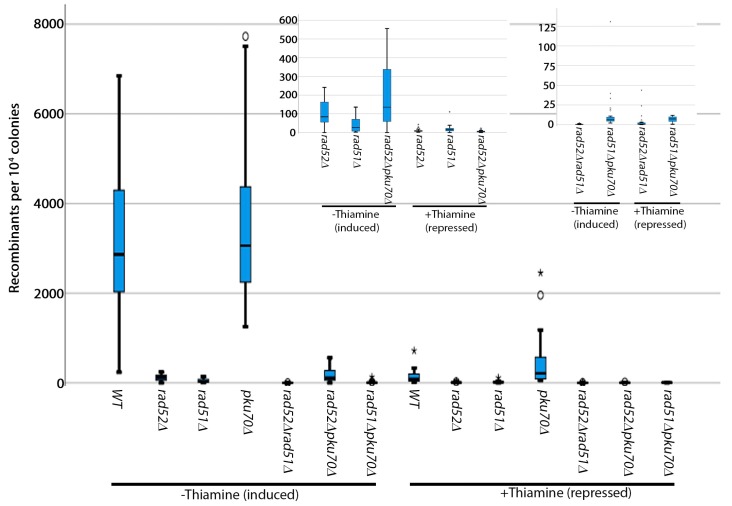
Genetic requirements for induced breaks. Box plots showing the *HO*-endonuclease-induced recombination frequency per 10^4^ colonies. Cells were grown on EMM-Uracil plates for 3–5 days at 32 °C. For clarity, insets are shown for strains with similar recombination frequencies.

Next, we validated the genetic requirements for induced breaks. Because the *HO* endonuclease is expressed from a plasmid behind the *nmt1* promoter, we used the following controls: 1) a control with thiamine to check whether the promoter is leaky and 2) a vector control which should give a frequency comparable to the spontaneous frequency (Figure 3, Supplementary Table S4). We found that recombinants appeared at about three orders of magnitude higher when the break was induced. The addition of thiamine drastically reduced the recombination frequency—albeit not to the spontaneous levels, suggesting that there is some promoter leakage. The vector control gives spontaneous level recombinants.

Both *rad52^+^* and *rad51^+^* are required for recombinants arising from induced breaks, suggesting that induced breaks are likely repaired through a crossover—either intrachromosomal or unequal sister chromatid exchange. As previously shown [38,39], *pku70*^+^ antagonizes recombination and, not unexpectedly, we also show that the deletion of *pku70^+^* increases recombination outcomes. However, the function of *pku70^+^* here is distinct from its function at spontaneous breaks, because it does not have the same phenotype as *rad51^+^*. The small increase in the frequency of recombinants in the absence of *pku70^+^* suggests that the cell attempts to repair some of the generated two-ended breaks through NHEJ, which does not result in a *ura4^+^* phenotype. Non-homologous end-joining and *pku70*^+^ have been previously shown to compete with recombination [29,38,39]. Because we select for *ura4^+^*, these cells will die. The requirement of *pku70^+^* for the induced breaks, but not *rad51^+^*, may be explained by the stage of the cell cycle where the breaks are repaired. We presume that spontaneous breaks are generated as a consequence of DNA replication and may be repaired in S-phase. *HO*-induced breaks cause a cell cycle delay due to activation of the *rad3^+^*-dependent checkpoint [29]. It is therefore possible that induced breaks pile up in G2, while spontaneous breaks are repaired in S-phase. These results suggest that the mechanism of repair of induced breaks is distinct from that of spontaneous breaks.

## 4. Conclusions

Here we describe an assay to study intra-chromosomal deletions arising at regions of non-tandem repeats and provide preliminary data that show that induced breaks have different genetic requirements than spontaneous breaks—in agreement with what has been previously shown. We believe that this assay will be an important tool in the field of DNA damage repair to study deletions.

It is also worth noting that some cell cycle regulators, such as CDKN2A, are inactivated in cancer cells by deletion of the entire gene rather than by point mutations [40,41]. This shows that these intra-chromosomal deletions could introduce enough genetic change in human cells that may cause cancer. Since there is enough conservation in repair genes between yeast and human cells, this assay could be used to leverage the powerful yeast genetics to identify the mechanisms of these intra-chromosomal deletions.

## Supplementary Materials

The following are available online at https://www.mdpi.com/2409-9279/2/3/74/s1, Figure S1: Analysis of Ura+His+ recombinants, Figure S2: Sensitivity of strains to MMS, Table S1: Strains used in this study, Table S2: Descriptive statistics for spontaneous breaks when cells were released in Edinburgh Minimal Media and platted on EMM-Uracil with Phloxin B, Table S3: Descriptive statistics for spontaneous breaks when cells were released in Edinburgh Minimal Media and platted on EMM-Uracil without Phloxin B, Table S4: Descriptive statistics for induced breaks when cells were released in Edinburgh Minimal Media.
