# Supplementary material for: An Assay to Study Intra-Chromosomal Deletions in Yeast
Source: Methods Protoc. 2019 Aug 26;2(3):74. doi: 10.3390/mps2030074 (PMC6789737; doi:10.3390/mps2030074)
Supplement: Supplementary file 1 [file mps-02-00074-s001.pdf]

Supplementary Material

Supplementary Figure Legends

Supplementary Figure S1. Analysis of Ura+His+ recombinants. A. Box-plot comparing the frequency of Ura+ and Ura+His+ recombinants. Note that the scale is logarithmic and that the frequency of the Ura+His+ is approximately three orders of magnitude lower. **B**. PCR across the *ura4*$^+$ locus with same primers form Figure 1C. Shown are pre-recombinants (e.g. intact *ura-his-ura*) and post recombinants Ura+ and Ura+His+. Only when the break is induced can Ura+His+ be recovered. **C.** PCR to check the his3+ locus on chromosome 2 in WT (*his3*$^+$), Pre (*ura4::ura'-his-'ura, his3-D1*) and induced recombinants Ura+His+. The top gel labeled "PCR across *his3*$^+$ locus" is PCR with primers upstream and downstream the his3$^+$ open reading frame. Note that the WT (*his3*$^+$) has a longer fragment than both pre and post recombinants which should be *his3-D1*. The *his3-D1* allele is a deletion of most of the *his3*$^+$ ORF. This indicates that the *his3-D1* locus has not been converted to *his3*$^+$. The bottom gel labeled "PCR within the *his3*$^+$ ORF" is PCR with primers within the *his3*$^+$ ORF. Note that all samples produce the same band indicating that they all have an intact *his3*$^+$ ORF.

Supplementary Figure S2. Sensitivity of strains to MMS. Strains of the indicated genotypes were grown in YES at 32°C overnight then 5X serial dilutions were spotted onto YES plates or YES+0.008%MMS. Plates were incubated at 32°C for 4 days. Note that both the *rad52Δ* and *rad51Δ* mutant strains remain sensitive indicating that they did not acquire a suppressor.

Supplementary Figure S1: Analysis of Ura4^{+}His3^{+} recombinants

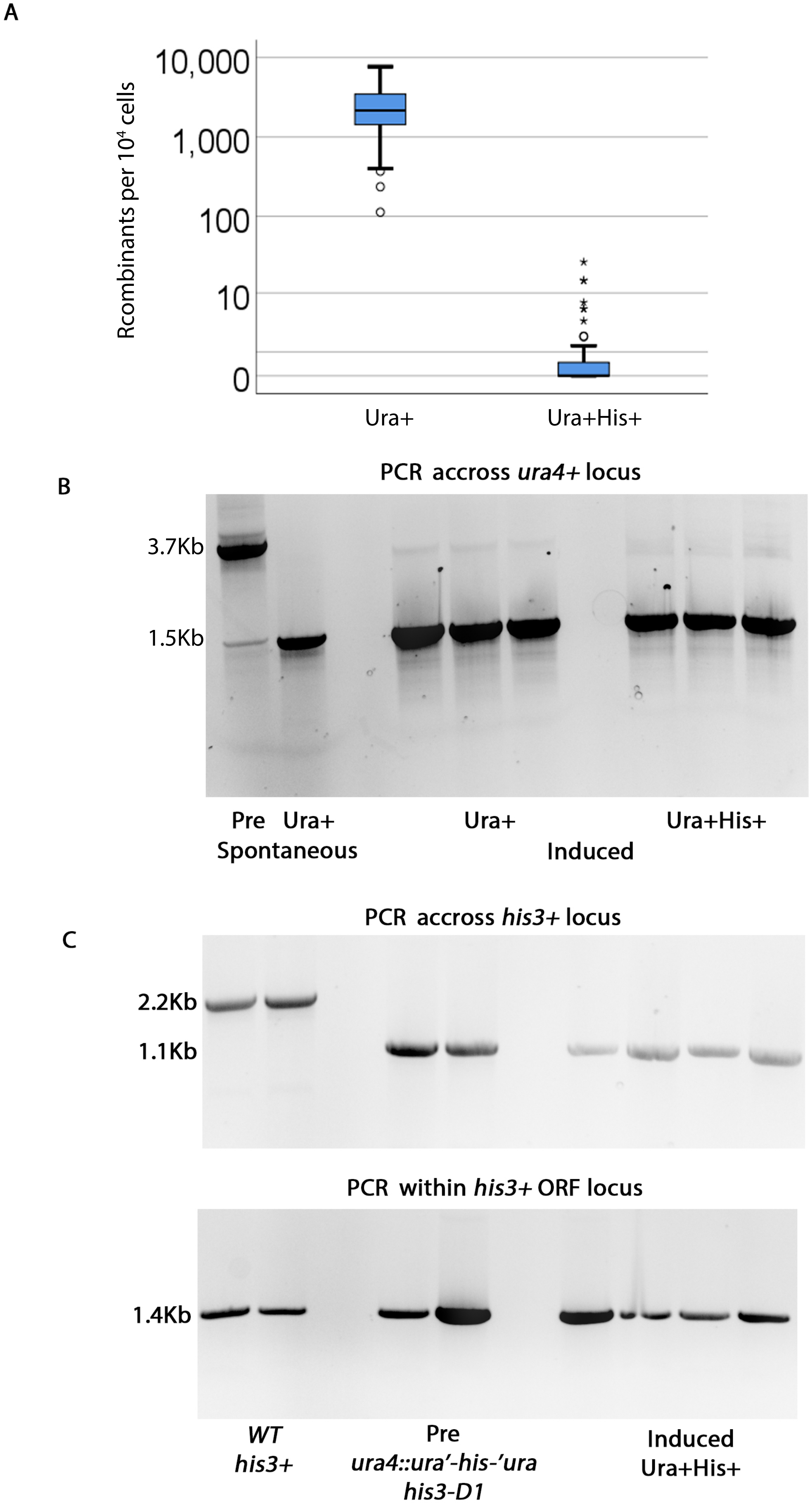

Supplementary Figure S2. Sensitivity of mutants to MMS

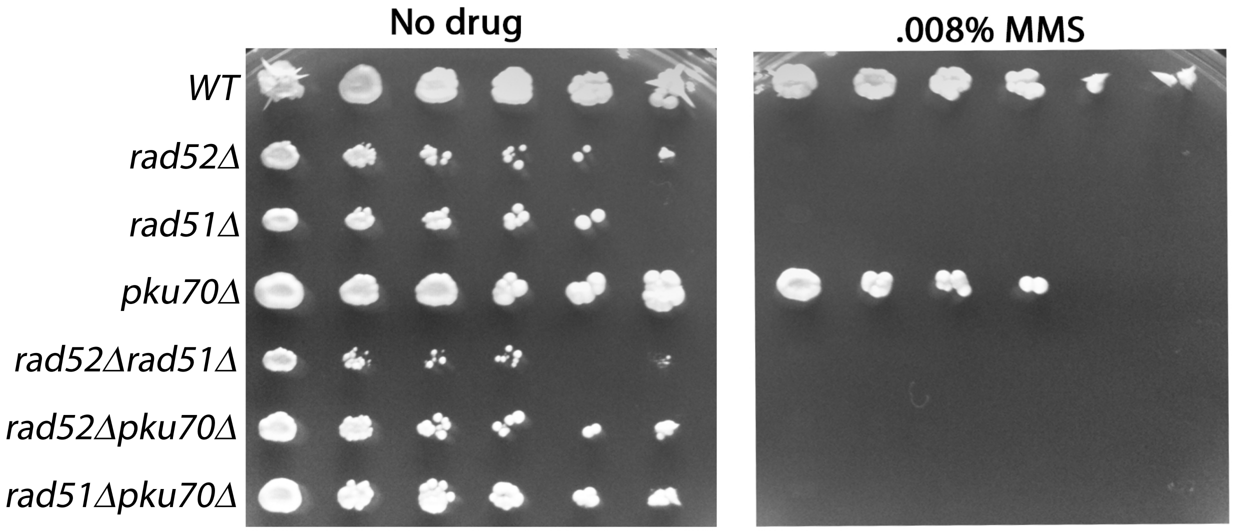

Supplementary Table S1. Strains used in this study.

Identifier	Genotype	Source
FY 1828	*h^+ his3-D1 leu1-32*	Forsburg
RCP 24	*h^+ ura4::ura4-his3-HO-ura4 his3-D1 leu1-32*	Forsburg
RCP 71	*h^- Δrad52::kanMX4-Bioneer ura4::ura4-his3-HO-ura4 his3-D1 leu1-32 ade6-M210/216?*	This Study
RCP 228	*h^- smt-0 Δrhp51::kanMX4-Bioneer ura4::ura4-his3-ura4 leu1-32 ade6-M216/210 his3-D1*	Forsburg
RCP 178	*h^- Δpku70::kanMX ura4::ura4-his3-HO-ura4 leu1-32 his3-D1 ade6-M210*	This Study
RCP 256	*h^+ Δrad52::kanMX4-Bioneer Δrad51::kanMX4-Bioneer ura4::ura4-his3-ura4 leu1-32 his3-D1 ade6-M216/210*	This Study
RCP 258	*h^- Δrad52::kanMX4-Bioneer Δpku70::kanR ura4::ura4-his3-HO-ura4 leu1-32 his3-D1 ade6-M210*	This Study
RCP 275	*h^- smt-0 Δrad51::kanMX4-Bioneer Δpku70::kanR ura4::ura4-his3-ura4 his3-D1 leu1-32 ade6-M216/210*	This Study
RCP 124	*h^+ ura4::ura4-his3-HO-ura4 his3-D1 leu1-32/ pREP81X-HO*	This Study
RCP 81	*h^- Δrad52::kanMX4-Bioneer ura4::ura4-his3-HO-ura4 his3-D1 leu1-32 ade6-M210/216 /pREP81X-HO*	This Study
RCP 267	*h^- smt-0 Δrad51::kanMX4-Bioneer ura4::ura4-his3-ura4 leu1-32 his3-D1 ade6-M216/210 /pREP81X-HO*	This Study
RCP 371	*h^- Δpku70::kanMX ura4::ura4-his3-HO-ura4 leu1-32 his3-D1 ade6-M210 /pREP81X-HO*	This Study
RCP 268	*h^+ Δrad52::kanMX4-Bioneer Δrad51::kanMX4-Bioneer ura4::ura4-his3-ura4 leu1-32 his3-D1 ade6-M216/210 /pREP81X-HO*	This Study
RCP 288	*h^- Δrad51::kanMX4-Bioneer Δpku70::kanMX ura4::ura4-his3-ura4 leu1-32 ade6-M216/210 ura4-D18 his3-D1 /pREP81X-HO*	This Study
RCP377	*h^- Δpku70::kanMX Δrad52::kanMX-bioneer ura4::ura4-his3-HO-ura4 leu1-32 his3-D1 ade6-M210 /pREP81X-HO*	This Study

Supplementary Table S2. Descriptive statistics for spontaneous breaks when cells were released in Edinburgh Minimal Media and platted on EMM-Uracil with Phloxin B. Recombinants per 10^5 cells.

	N	Mean		Std. Deviation
	Statistic	Statistic	Std. Error	Statistic
WT	36	30.70	4.20	25.20
Δrad52	30	14.98	3.61	19.77
Δrad51	19	175.14	22.64	98.69
Δpku70	18	146.32	34.41	146.00
Δrad52Δrad51	24	18.82	5.41	26.48
Δrad52Δpku70	25	5.65	1.24	6.18
Δrad51Δpku70	17	192.82	58.79	242.39

Supplementary Table S3. Descriptive statistics for spontaneous breaks when cells were released in Edinburgh Minimal Media and plated on EMM-Uracil without Phloxin B. Recombinants per 10^5 cells.

	N	Mean		Std. Deviation
	Statistic	Statistic	Std. Error	Statistic
WT	20	24.94	8.40	37.58
Δrad52	29	14.74	3.41	18.34
Δrad51	10	156.81	27.77	87.81
Δpku70	18	154.81	23.48	99.63
Δrad52Δrad51	15	19.72	4.08	15.81
Δrad52Δpku70	25	4.89	0.74	3.69
Δrad51Δpku70	12	157.03	42.46	147.07

Supplementary Table S4. Descriptive statistics for induced breaks when cells were released in Edinburgh Minimal Media. Recombinants per 10^4 cells.

	N	Mean		Std. Deviation
	Statistic	Statistic	Std. Error	Statistic
WT + pREP81X-HO w/ Thiamine	26	154.64	33.34	170.01
Δrad52 + pREP81X-HO w/ Thiamine	50	10.77	1.06	7.52
Δrad51 + pREP81X-HO w/ Thiamine	33	18.76	3.36	19.31
Δpku70 + pREP81X-HO w/ Thiamine	35	370.07	92.24	545.70
Δrad52Δrad51 + pREP81X-HO w/ Thiamine	29	3.66	1.68	9.04
Δrad52Δpku70 + pREP81X-HO w/ Thiamine	30	5.28	0.83	4.53
Δrad51Δpku70 + pREP81X-HO w/ Thiamine	29	6.84	0.57	3.07
WT + pREP81X-HO w/o Thiamine	47	3045.98	259.99	1782.37
Δrad52 + pREP81X-HO w/o Thiamine	34	99.87	10.95	63.84
Δrad51 + pREP81X-HO w/o Thiamine	46	35.12	5.27	35.76
Δpku70 + pREP81X-HO w/o Thiamine	26	3684.68	344.18	1754.99
Δrad52Δrad51 + pREP81X-HO w/o Thiamine	39	0.46	0.079	0.49
Δrad52Δpku70 + pREP81X-HO w/o Thiamine	36	235.55	29.21	175.24
Δrad51Δpku70 + pREP81X-HO w/o Thiamine	28	13.54	4.7	24.63
WT + pREP81X-Vector w/o Thiamine	50	5.67	1.27	8.95
Δrad52 + pREP81X-Vector w/o Thiamine	28	0.79	0.20	1.06
Δrad51 + pREP81X-Vector w/o Thiamine	19	7.68	2.72	11.85
Δpku70 + pREP81X-Vector w/o Thiamine	N/A			
Δrad52Δrad51 + pREP81X-Vector w/o Thiamine	20	0.14	0.02	0.11
Δrad52Δpku70 + pREP81X-Vector w/o Thiamine	35	0.77	0.24	1.41
Δrad51Δpku70 + pREP81X-Vector w/o Thiamine	20	2.84	0.84	3.77
